# Role of CTCF in the regulation of microRNA expression

**DOI:** 10.3389/fgene.2012.00186

**Published:** 2012-09-25

**Authors:** Yoshimasa Saito, Hidetsugu Saito

**Affiliations:** Division of Pharmacotherapeutics, Faculty of Pharmacy, Keio UniversityTokyo, Japan

**Keywords:** microRNA, CTCF, cancer cell, embryonic stem cell, *miR-125b1*, *miR-375*, *miR-290* cluster

## Abstract

MicroRNAs (miRNAs) are small non-coding RNAs that regulate expression of various target genes. miRNAs are expressed in a tissue-specific manner and play important roles in cell proliferation, apoptosis, and differentiation. Epigenetic alterations such as DNA methylation and histone modification are essential for chromatin remodeling and regulation of gene expression including miRNAs. The CCCTC-binding factor, CTCF, is known to bind insulators and exhibits an enhancer-blocking and barrier function, and more recently, it also contributes to the three-dimensional organization of the genome. CTCF can also serve as a barrier against the spread of DNA methylation and histone repressive marks over promoter regions of tumor suppressor genes. Recent studies have shown that CTCF is also involved in the regulation of miRNAs such as *miR-125b1*, *miR-375*, and the *miR-290* cluster in cancer cells and stem cells. *miR-125b1* is a candidate of tumor suppressor and is silenced in breast cancer cells. On the other hand, *miR-375* may have oncogenic function and is overexpressed in breast cancer cells. CTCF is involved in the regulation of both *miR-125b1* and *miR-375*, indicating that there are various patterns of CTCF-associated epigenetic regulation of miRNAs. CTCF may also play a key role in the pluripotency of cells through the regulation of *miR-290* cluster. These observations suggest that CTCF-mediated regulation of miRNAs could be a novel approach for cancer therapy and regenerative medicine.

## Introduction

MicroRNAs (miRNAs) are small non-coding RNAs that regulate various target genes and play important roles in cell proliferation, apoptosis, and differentiation. One of the important mechanisms of miRNA expression is epigenetic alteration such as DNA methylation and histone modification. The CCCTC-binding factor, CTCF, is known to bind insulators and exhibits an enhancer-blocking and barrier function, and more recently, it also contributes to the three-dimensional organization of the genome. Although, there are a number of studies describing regulation of miRNA expression including epigenetic alterations, only a few studies have reported the association between miRNA expression and CTCF. In this report, we review recent studies regarding miRNAs and CTCF, and discuss about roles of CTCF in the regulation of miRNA expression.

## miRNA

miRNAs are ~22 nucleotide (nt) non-coding RNAs that can post-transcriptionally downregulate the expression of various target genes. Currently, ~1500 human miRNAs have been identified in the human genome, and each miRNA potentially controls hundreds of target genes. In animals, miRNA genes are generally transcribed by RNA polymerase II (pol II) to form primary transcripts (pri-miRNAs). Pol II transcribed pri-miRNAs are capped with 7-methylguanosine and are polyadenylated. The nuclear RNase III enzyme Drosha and its co-factor DGCR8 process pri-miRNAs into ~60 nt precursor miRNAs (pre-miRNAs), which form an imperfect stem-loop structure. Pre-miRNAs are transported into the cytoplasm by exportin 5 and are subsequently cleaved by Dicer into mature miRNAs which are then loaded into the RNA-induced silencing complex (RISC). The miRNA/RISC complex downregulates specific gene products by translational repression via binding to partially complementary sequences in the 3′ untranslated regions of the target mRNAs or by directing mRNA degradation via binding to perfectly complementary sequences. miRNAs are expressed in a tissue-specific manner and play important roles in metabolism, proliferation, apoptosis, and differentiation. Moreover, recent studies have shown a link between aberrant expression of miRNAs and the development of cancer (Calin and Croce, 2007; Cho, 2007; Saito et al., 2009).

## Epigenetic regulation of miRNA expression

Since miRNAs can have large-scale effects through regulation of a variety of genes during mammalian development and carcinogenesis, an understanding of the regulatory mechanisms controlling miRNA expression is important. There are several reports of transcription factors binding to the promoter regions of specific miRNA genes and activating the transcription of pri-miRNAs, resulting in increased expression of mature miRNAs. *c-Myc* binds to the regulatory region of the *miR-17-92* cluster and increased expression of *c-Myc* leads to the activation of the miRNAs in the cluster (O'Donnell et al., 2005).

Epigenetic alterations such as DNA methylation and histone modification play critical roles in chromatin remodeling and regulation of gene expression in mammalian development and in human diseases. Many miRNAs are expressed in a tissue- and tumor-specific manner, implying that some miRNAs are subject to epigenetic control. We have shown that *miR-127*, which is embedded in a CpG island, is strongly induced by treatment with DNA methylation inhibitors and histone deacetylase inhibitors, indicating that some miRNA genes are controlled by epigenetic alterations in their promoter regions and can be activated by chromatin modifying drugs (Saito et al., 2006, 2009). Lujambio et al. (2007) compared miRNA expression profiling between the wild-type HCT116 colon cancer cell line and HCT116 after genetic disruption of both *DNA methyltransferase (DNMT) 1* and *DNMT3b* (DKO cells). They found that 18 out of 320 miRNAs are significantly upregulated in DKO cells. In particular, *miR-124a* is silenced by its own CpG island hypermethylation in human tumors, but can be activated by inhibition of DNA methylation. They also demonstrated that the oncogene *CDK6* is a target of *miR-124a* and that epigenetic silencing of *miR-124a* in cancer cells modulates *CDK6* activity. It has been reported that *miR-9-1* and *miR-9-3* are potential tumor suppressor miRNAs and are inactivated by epigenetic mechanisms in human cancers (Lehmann et al., 2008; Lujambio et al., 2008). *miR-34a* was identified as a target of p53 and induces a G(1) cell cycle arrest, senescence and apoptosis (He et al., 2007; Tazawa et al., 2007). *miR-34a* expression is silenced in several types of cancer including pancreatic cancer due to aberrant CpG methylation of its promoter. Re-expression of *miR-34a* in a pancreatic carcinoma cell line induced senescence and cell cycle arrest at least in part by targeting *CDK6*, indicating that *miR-34a* represents a tumor suppressor gene which is inactivated by CpG methylation in pancreatic cancer (Lodygin et al., 2008). *miR-34b* and *miR-34c* are also reported to be silenced by aberrant CpG island methylation in colorectal cancer (Toyota et al., 2008). Thus, a number of miRNAs are under epigenetic control and disruption of DNA methylation patterns and histone modification in the promoter regions of miRNAs might be associated with cancer development (Esteller, 2011).

The CCCTC-binding factor, CTCF, is known to bind insulators and exhibits an enhancer-blocking function. CTCF can also serve as a barrier against the spread of DNA methylation and histone repressive marks over promoter regions of tumor suppressor genes (Recillas-Targa et al., 2011). CTCF is a highly conserved multifunctional zinc finger protein involved in transcriptional repression and activation, insulation, epigenetic events such as imprinting of the *H19/IGF2* locus, and X-inactivation, and which binds preferentially to unmethylated DNA (Filippova, 2008; Phillips and Corces, 2009). Moreover, CTCF play important roles during carcinogenesis: epigenetic silencing of tumor suppressor genes such as *p16* and *Rb* (De La Rosa-Velazquez et al., 2007; Witcher and Emerson, 2009), apoptosis of breast cancer cells (Docquier et al., 2005), and regulation of important tumor suppressor genes such as *p53* (Recillas-Targa et al., 2011; Saldana-Meyer and Recillas-Targa, 2011). These findings suggest that CTCF may be involved in epigenetic regulation of non-coding RNAs including miRNAs as well as coding RNAs.

## Disruption of CTCF binding at the *miR-125b1* CpG island in human cancers

Recent studies have reported that expression of *miR-125b* is downregulated in various human cancers including glioblastoma, prostate cancer, ovarian cancer, and breast cancer (Scott et al., 2007; Zhang et al., 2011). In addition, *miR-125b* suppresses oncogenes such as EST1, ERBB2, ERBB3, and Bak1 as its targets, suggesting that *miR-125b* functions as a tumor suppressor. DNA hypermethylation at the CpG island of *miR-125b* was observed in cell lines and in tissue samples from patients with breast cancer (Zhang et al., 2011).

**Figure 1:**
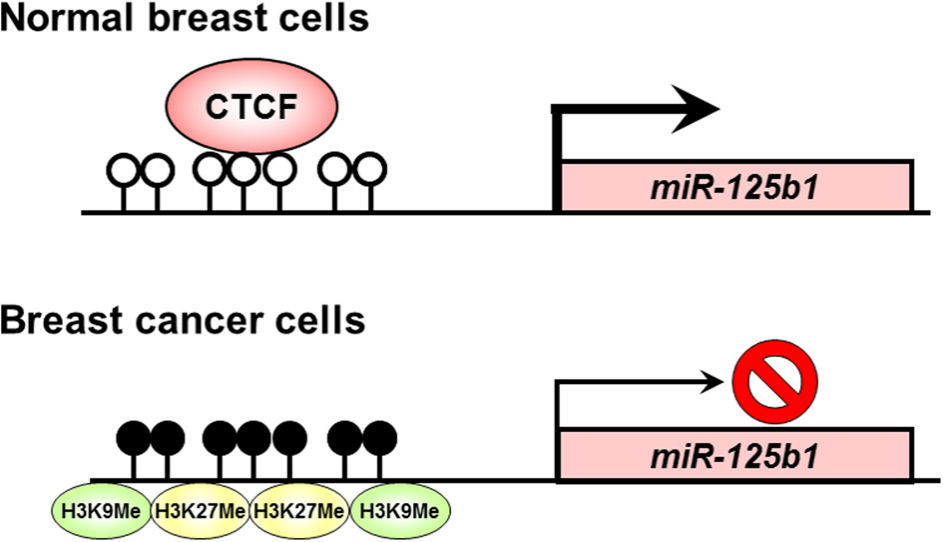
**Disruption of CTCF binding at the *miR-125b1* CpG island in human cancers.** In normal breast cells, CTCF might prevent the recruitment of epigenetic silencing components, such as DNA methylation and repressive histone modifications, and also favors an open chromatin structure. Meanwhile, in breast cancer cells, the loss of CTCF is associated with CpG island methylation and the gain of repressive histone modifications such as histone H3 K9 trimethylation and histone H3 K27 trimethylation. Open circle, unmethylated DNA; filled circle, methylated DNA; H3K9Me, histone H3 K9 methylation; H3K27Me, histone H3 K27 methylation.

Soto-Reyes et al. (2012) investigated epigenetic alterations such as DNA methylation and histone modification, and association of CTCF at the locus of *miR-125b1* in breast cancer cells. They found aberrant DNA methylation of the *miR-125b1* CpG island and that disruption of CTCF binding correlated with incorporation of repressive histone modifications such as histone H3 lysine 9 (K9) trimethylation and histone H3 K27 trimethylation in cancer cells. In normal breast cells expressing *miR-125b1*, CTCF might prevent the recruitment of epigenetic silencing components, such as DNA methylation and repressive histone modifications, and also favors an open chromatin structure. In breast cancer cells, the loss of CTCF is associated with CpG island methylation and the gain of repressive histone modifications such as histone H3 K9 trimethylation and histone H3 K27 trimethylation. Disruption of CTCF binding at CpG island induces silencing of *miR-125b1* expression (Figure 1). These findings suggest that CTCF plays an important role in the regulation of the tumor suppressor *miR-125b1* in cooperation with DNA methylation and histone modification in breast cancer cells. A recent study has also demonstrated that *miR-125b1* can be silenced by DNA methylation, which may lead to activation of the ETS1 proto-oncogene and a worse prognosis in breast cancer patients (Zhang et al., 2011). Reactivation of the tumor suppressor *miR-125b1* by epigenetic therapy using DNA methylation inhibitors may have clinical promise for the treatment of breast cancer patients.

## Role of CTCF in the regulation of *miR-375* expression in breast cancer cells

**Figure 2:**
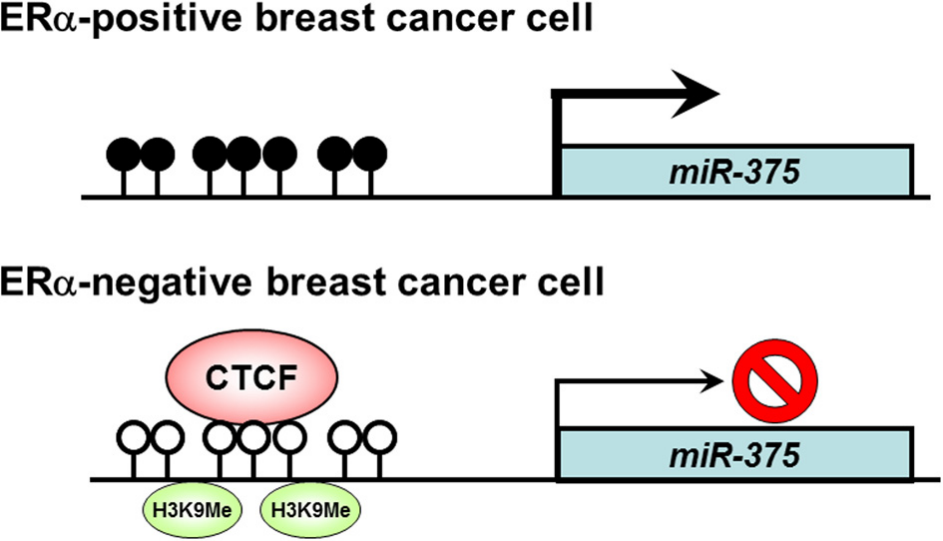
**Role of CTCF in the regulation of *miR-375* expression in breast cancer cells.** In ERα-positive breast cancer cells, DNA hypermethylation is observed in the CpG island of the *miR-375* gene, and *miR-375* expression is activated. On the other hand, in ERα-negative breast cancer cells, DNA hypomethylation and histone H3 K9 dimethylation are observed, resulting in silencing of *miR-375* expression by binding of CTCF in the CpG island. Open circle, unmethylated DNA; filled circle, methylated DNA; H3K9Me, histone H3 K9 methylation.

Breast cancer is the leading cause of cancer death in women worldwide. Estrogen receptor α (ER α) upregulation causes abnormal cell proliferation in approximately 70% of breast cancers (Shoker et al., 1999; Vargo-Gogola and Rosen, 2007). A recent study has reported that *miR-375* is overexpressed in ERα-positive breast cancer cell lines and plays an important role in cell proliferation (de Souza Rocha Simonini et al., 2010). There are CpG islands in the upstream region of the *miR-375* gene. DNA hypermethylation is observed in the CpG island of ERα-positive breast cancer cells showing high expression of *miR-375*, whereas DNA hypomethylation and histone H3 K9 dimethylation are observed in the CpG islands of ERα-negative breast cancer cells. CTCF binds to unmethylated DNA in the CpG islands of ERα-negative cells and induces silencing of *miR-375* expression. These findings suggest that overexpression of *miR-375* is caused by dissociation of CTCF from the CpG island of *miR-375* gene via loss of epigenetic marks including local DNA hypomethylation and histone H3 K9 dimethylation (de Souza Rocha Simonini et al., 2010) (Figure 2). It has been shown that *miR-375* suppresses *Ras dexamethasone-induced 1* (*RASD1*) as its potential target, and RASD1 can suppress the growth of breast cancer cells and down-regulate ERα expression (Vaidyanathan et al., 2004; de Souza Rocha Simonini et al., 2010). Thus the modulation of ERα expression by *miR-375* is achieved through the repression of RASD1. These observations provide a possibility that inhibition of *miR-375* could be a novel clinical approach for the treatment of ERα-positive breast cancer.

## CTCF modulates expression of the early embryonic miRNA cluster

Human embryonic stem cells (ESCs) are derived from the inner cell mass of the human blastocyte and can be kept in an undifferentiated, self-renewing state indefinitely. ESCs have the advantage of being pluripotent, which endows them with the ability to differentiate into virtually every cell type in the human body. Thus, ESCs have gained popularity as a potentially ideal cell candidate for regenerative medicine. The early embryonic miRNA cluster (EEmiRC) has been identified in ESCs of mammals, and shows a remarkable cross-eutherian species conservation at the levels of both pre-miRNA hairpins and the core-promoter region (Houbaviy et al., 2003, 2005). EEmiRC encodes 7 miRNAs (*miR-290*, -*291a*, -*292*, -*291b*, -*293*, -*294* and -*295*), which have been labeled as ESC-specific/pluripotency-associated miRNAs controlling cell-cycle progression, proliferation, and DNA methylation in undifferentiated/pluripotent cells. Therefore, understanding the biology of ESCs requires detailed knowledge of the mechanisms regulating EEmRC expression.

Little is known about the molecular mechanisms underlying the regulation of the EEmiRC expression. Recent studies have showed that the sequences upstream to the EEmiRC promoter contains active binding sites for Nanog, Oct3/4, Sox2, Tcf3, c-Myc, and 4n-Myc. Histone H3 K4 trimethylation and histone H3 K27 trimethylation were observed in ESCs and in differentiated cells, respectively (Chen et al., 2008; Judson et al., 2009). However, attempts to activate EEmiRC expression by ectopic expression of these individual transcriptional factors in fibroblasts were unsuccessful, suggesting that EEmiRC expression is under epigenetic control (Judson et al., 2009). Tata et al. (2011) identified a 332-bp intragenic enhancer (IE) region within the EEmiRC, which is able to modulate the transcription of the mouse EEmiRC locus. These miRNAs involve pluripotency factors and epigenetic mechanisms in pluripotent and differentiated cells. The results of chromatin immunoprecipitation (ChIP) assays demonstrated that the level of occupancy of Oct3/4, Sox2, and CTCF in this region gradually and dramatically decreased during ESC differentiation, suggesting a functional role for these transcription factors in regulating EEmiRC expression. This IE also contains a CpG island showing a differential pattern of DNA and histone methylation marks during differentiation of ESCs. Since, *miR-290* cluster miRNAs have been shown to suppress Rbl2 as their target and Rbl2 modulates DNMTs (Benetti et al., 2008; Sinkkonen et al., 2008), EEmiRC may comprise a feedback loop with DNMTs. These findings indicate that this region plays a critical role in the regulation of EEmiRC expression, presumably through binding of transcription modulators such as Oct3/4, Sox2, and CTCF. Cohesin is a DNA-binding protein complex that is essential for sister chromatid cohesion and facilitates the repair of damaged DNA. Recent experiments have revealed that cohesin binds to the same sites in mammalian genomes as CTCF and cooperates with CTCF in regulating gene expression (Herold et al., 2012). Epigenetic effectors including CTCF and cohesin may modulate the pluripotency of cells through the regulation of *miR-290* cluster.

## Perspectives and conclusion

Table 1 shows a summary of the association between miRNAs and CTCF. These findings indicate that the insulator protein CTCF plays various roles in the regulation of miRNAs such as *miR-125b1*, *miR-375*, and the *miR-290* cluster during mammalian development and carcinogenesis. *miR-125b1* is a candidate of tumor suppressor and is silenced in breast cancer cells. On the other hand, *miR-375* may have oncogenic function and is overexpressed in breast cancer cells. CTCF is involved in the regulation of both *miR-125b1* and *miR-375*, indicating that there are various patterns of CTCF-associated epigenetic regulation of miRNAs. CTCF-mediated regulation of these miRNAs may provide a novel therapeutic approach for breast cancer. CTCF may also play a key role in the pluripotency of cells through the regulation of *miR-290* cluster. Since, the link between miRNAs and CTCF has only just begun to be understood, other miRNA genes regulated by CTCF will be identified. Further studies are necessary to investigate whether CTCF-mediated regulation of miRNAs could be a novel approach for cancer therapy and regenerative medicine.

**Table 1:** **miRNAs associated with CTCF**.

**miRNA**	**Expression**	**Target genes**	**Association with CTCF**	**References**
*miR-125b1*	Decreased in human cancers including glioblastoma, prostate cancer, ovarian cancer and breast cancer	EST1, ERBB2, ERBB3, Bak1	In breast cancer cells, disruption of CTCF binding at the *miR-125b1* CpG island correlated with DNA methylation and methylation of histone H3K9 and K27 induces silencing of *miR-125b1* expression	Scott et al., 2007; Zhang et al., 2011; Soto-Reyes et al., 2012
*miR-375*	Overexpressed in ERα-positive breast cancer cells	RASD1	In ERα-positive breast cancer cells, *miR-375* overexpression was caused by dissociation of CTCF from the *miR-375* promoter via loss of epigenetic marks including local DNA hypomethylation and histone H3 K9 methylation	de Souza Rocha Simonini et al., 2010
*miR-290* cluster	*miR-290* cluster (*miR-290, -291a, -292, -291b, -293, -294 and -295*) have been identified as ESC-specific/pluripotency-associated miRNAs	Rbl2	CTCF binds to intragenic enhancer region within the early embryonic miRNA cluster (EEmiRC) and modulates the expression of the EEmiRC	Houbaviy et al., 2003, 2005; Benetti et al., 2008; Chen et al., 2008; Sinkkonen et al., 2008; Judson et al., 2009; Tata et al., 2011

### Conflict of interest statement

The authors declare that the research was conducted in the absence of any commercial or financial relationships that could be construed as a potential conflict of interest.

## Abbreviations

miRNA: microRNA
RISC: RNA-induced silencing complex
ERα: estrogen receptor α
ESC: embryonic stem cell
EEmiRC: early embryonic miRNA cluster
IE: intragenic enhancer.
